# Prognostic value of the atherogenic index of plasma combined with lactate-to-albumin ratio for in-hospital mortality in patients with acute myocardial infarction complicated by cardiogenic shock

**DOI:** 10.3389/fcvm.2026.1816471

**Published:** 2026-09-14

**Authors:** Yinsheng Jin, Kai Yang, Lingling Yao

**Affiliations:** 1 Department of Cardiac Care Unit (CCU), Renmin Hospital, Hubei University of Medicine, Shiyan, Hubei, China; 2 Emergency Department, Renmin Hospital, Hubei University of Medicine, Shiyan, Hubei, China

**Keywords:** acute myocardial infarction, atherogenic index of plasma, cardiogenic shock, lactate-to-albumin ratio, nomogram, prognosis

## Abstract

**Background:**

Acute myocardial infarction complicated by cardiogenic shock (AMICS) carries high in-hospital mortality. We evaluated the combined prognostic value of the Atherogenic Index of Plasma (AIP), reflecting lipid metabolism, and the Lactate-to-Albumin Ratio (LAR), reflecting tissue hypoperfusion and inflammation, in AMICS.

**Methods:**

This retrospective cohort included 207 consecutive patients with AMICS between January 2023 and January 2025, classified as survivors (*n* = 174) or non-survivors (*n* = 33). Admission clinical, laboratory, echocardiographic, and treatment variables were collected. Least Absolute Shrinkage and Selection Operator (LASSO) regression selected candidate predictors; multivariable logistic regression identified independent predictors and generated a nomogram. Performance was assessed using the area under the receiver operating characteristic curve (AUC), calibration metrics, bootstrap internal validation, and decision curve analysis.

**Results:**

Non-survivors had higher AIP (0.33 ± 0.23 vs. 0.06 ± 0.30) and LAR than survivors (*P* < 0.05). LASSO identified six candidate predictors. Mechanical ventilation (OR = 10.10, 95% CI: 3.09–33.09, *P* < 0.001), LVEF <40% (OR = 3.49, 95% CI: 1.23–9.90, *P* = 0.019), AIP (OR = 4.30 per SD increase, *P* < 0.001), and LAR (OR = 1.88 per SD increase, *P* = 0.005) were independently associated with in-hospital mortality. The combined model showed good-to-excellent internal discrimination (AUC = 0.909, bootstrap 95% CI: 0.853–0.957), exceeding AIP (AUC = 0.777) or LAR (AUC = 0.735) alone. Calibration was acceptable (Hosmer–Lemeshow *P* = 0.853; Brier score = 0.077, bootstrap 95% CI: 0.052–0.103). The model underwent internal validation only and should be considered hypothesis-generating pending external validation.

**Conclusion:**

AIP and LAR were independently associated with in-hospital mortality and may reflect complementary lipid-metabolic and hypoperfusion-inflammatory pathways. The nomogram combines these biomarkers with hemodynamic and respiratory severity parameters and may support early risk stratification, but requires external multicenter validation before routine clinical use.

## 1 Introduction

Acute myocardial infarction (AMI) complicated by cardiogenic shock (CS) represents the most severe clinical manifestation of acute coronary syndrome, characterized by profound cardiac pump failure leading to systemic hypoperfusion. Despite advances in early revascularization strategies and mechanical circulatory support, AMICS remains associated with unacceptably high morbidity and mortality, with short-term mortality rates plateauing between 40% and 50% in contemporary registries (1–3). Specifically, real-world data from recent multicenter registries continue to report in-hospital mortality rates as high as 57% (4), and 30-day mortality remains approximately 40%–45% despite widespread adoption of primary percutaneous coronary intervention (5). The complexity of AMICS pathophysiology extends beyond simple hemodynamic failure; it involves a systemic inflammatory response syndrome (SIRS) and profound metabolic derangements that perpetuate multi-organ dysfunction, necessitating a multitarget therapeutic approach (6). Therefore, early and accurate risk stratification is paramount to guide aggressive therapeutic interventions and improve survival outcomes, underscoring the need for validated clinical prediction models (7).

Traditionally, risk stratification in acute myocardial infarction complicated by cardiogenic shock (AMICS) has relied on scoring systems such as the IABP-SHOCK II and CardShock scores, which primarily utilize demographic, hemodynamic, and basic laboratory parameters (8). These scores remain valuable for early mortality prediction, including in contemporary cohorts and across different treatment strategies (9). However, these scores may not fully capture the distinct metabolic and inflammatory phenotypes that contribute to refractory shock, a limitation highlighted by studies investigating biomarkers of specific pathways like glucagon-like peptide-1 (GLP-1) (10). Recently, there has been a paradigm shift towards integrating composite biomarkers that reflect overlapping pathological pathways.

Disorders of lipid metabolism are not only fundamental to the pathogenesis of atherosclerosis but also play a critical role in the acute stress response. The Atherogenic Index of Plasma (AIP), calculated as the logarithm of the molar ratio of triglycerides (TG) to high-density lipoprotein cholesterol (HDL-C) [log(TG/HDL-C)], has emerged as a comprehensive marker of plasma atherogenicity and is strongly associated with atherosclerotic burden (11). Elevated AIP is closely associated with a more atherogenic lipoprotein profile, including small dense LDL particles, and insulin resistance (12, 13). Recent evidence robustly suggests that AIP predicts adverse cardiovascular events in patients with coronary artery disease (14). Its prognostic utility extends to heart failure populations, including short-term mortality in acute decompensated heart failure and major adverse cardiovascular events in ischemic heart failure (15, 16). However, its prognostic utility specifically in the hyperacute, catabolic state of AMICS remains unknown.

The interplay between tissue hypoxia and inflammation is a hallmark of fatal cardiogenic shock (CS) (17, 18). Blood lactate serves as a cornerstone for assessing systemic hypoperfusion in critical care (19), while serum albumin acts as a negative acute-phase protein and a well-established marker of nutritional status (20–22). The Lactate-to-Albumin Ratio (LAR) integrates these two pathophysiological dimensions, providing a more stable and sensitive prognostic index than either parameter alone. LAR has demonstrated robust prognostic value for mortality in patients with sepsis and septic shock (23–25) as well as in those following cardiac arrest (26–28). Furthermore, emerging evidence supports its utility in risk stratification for patients with severe heart failure (29–31).

To our knowledge, the combined prognostic value of AIP and LAR has not been evaluated specifically in AMICS. Accordingly, the novelty of the present study lies in their joint evaluation within an AMICS-specific prediction model rather than in the assessment of either biomarker individually. We hypothesize that a “Metabolic-Inflammatory” axis, represented by these two composite biomarkers, significantly influences AMICS outcomes. This study aims to investigate the independent association of AIP and LAR with in-hospital mortality in AMICS patients and to develop a novel nomogram integrating these biomarkers to assist clinicians in early risk assessment.

## 2 Methods

### 2.1 Study design and population

This retrospective observational cohort study was conducted at the Coronary Care Unit (CCU) of Renmin Hospital, Hubei University of Medicine, China. We consecutively screened patients admitted with a primary diagnosis of acute myocardial infarction (AMI) complicated by cardiogenic shock (CS) between January 2023 and January 2025. The study protocol adhered to the principles of the Declaration of Helsinki and was approved by the Ethics Committee of Renmin Hospital, Hubei University of Medicine. The requirement for informed consent was waived due to the retrospective nature of the study, and all patient data were anonymized prior to analysis. The reporting of this study follows the Strengthening the Reporting of Observational Studies in Epidemiology (STROBE) guidelines (32).

The inclusion criteria were defined as follows: (1) Confirmed diagnosis of AMI according to the “Fourth Universal Definition of Myocardial Infarction” (33), characterized by a rise and/or fall of cardiac troponin (cTn) with at least one value above the 99th percentile upper reference limit, accompanied by clinical evidence of acute myocardial ischemia (symptoms, new ischemic ECG changes, development of pathological Q waves, or imaging evidence of new loss of viable myocardium); (2) Diagnosis of Cardiogenic Shock (CS) upon admission or within 24 h of hospitalization, defined by the following criteria (34): systolic blood pressure (SBP) <90 mmHg for >30 min or the need for vasopressors to maintain SBP >90 mmHg; signs of end-organ hypoperfusion (urine output <30 mL/h, cold extremities, altered mental status); and biochemical evidence of tissue hypoxia (serum lactate >2.0 mmol/L). Because SCAI shock stages were not prospectively recorded in the electronic medical record, SCAI staging was not used as an inclusion criterion or as a stratification variable in the primary analysis; this limitation is explicitly acknowledged in the Discussion.

We excluded patients based on the following criteria: (1) CS caused by non-AMI etiologies (e.g., fulminant myocarditis, severe valvular disease, or cardiomyopathy); (2) Out-of-hospital cardiac arrest (OHCA) with return of spontaneous circulation (ROSC) but severe hypoxic brain injury preventing neurological assessment; (3) Concomitant life-limiting comorbidities, including advanced malignancy with a life expectancy <1 year, end-stage renal disease (ESRD) requiring chronic dialysis, or severe hepatic failure (Child-Pugh Class C); (4) Acute confounding conditions such as acute stroke, massive pulmonary embolism, or septic shock; (5) Incomplete key clinical or laboratory data (missing >20% of variables).

### 2.2 Data collection and definitions

Demographic and clinical data were extracted from the electronic medical record system by two independent investigators who were blinded to the study outcomes. Baseline characteristics included age, gender, body mass index (BMI), history of hypertension, diabetes mellitus, prior coronary artery disease (CAD), chronic heart failure (CHF), and smoking status. Clinical parameters recorded at admission comprised heart rate (HR), SBP, diastolic blood pressure (DBP), and Glasgow Coma Scale (GCS) score.

Laboratory investigations were performed on the first available venous or arterial blood samples obtained immediately upon admission to the Emergency Department or CCU. These admission samples were used for biomarker calculation and were generally obtained before PCI, mechanical circulatory support, CRRT, or mechanical ventilation whenever clinically feasible; however, emergency vasopressor therapy may have been initiated before sampling in some unstable patients. Hematological and biochemical parameters analyzed included complete blood count (white blood cell count, hemoglobin, platelet count), renal function (serum creatinine, blood urea nitrogen), liver function [alanine aminotransferase (ALT), aspartate aminotransferase (AST), albumin], cardiac biomarkers [high-sensitivity cardiac troponin I (hs-cTnI), N-terminal pro-B-type natriuretic peptide (NT-proBNP)], lipid profile [total cholesterol, triglycerides (TG), low-density lipoprotein cholesterol (LDL-C), high-density lipoprotein cholesterol (HDL-C)], and inflammatory markers [high-sensitivity C-reactive protein (hs-CRP)]. Arterial blood gas (ABG) analysis provided pH and lactate levels. Echocardiographic data, specifically the first transthoracic left ventricular ejection fraction (LVEF), were obtained within 24 h of admission, usually after initial stabilization in the CCU. Treatment variables, including the use of mechanical ventilation (MV), intra-aortic balloon pump (IABP), extracorporeal membrane oxygenation (ECMO), continuous renal replacement therapy (CRRT), vasoactive drugs, defibrillation, and percutaneous coronary intervention (PCI), were also documented.

The primary outcome of the study was all-cause in-hospital mortality. The Atherogenic Index of Plasma (AIP) was calculated as the base-10 logarithm of the molar ratio of triglycerides to HDL-cholesterol: AIP = log(TG/HDL-C). The Lactate-to-Albumin Ratio (LAR) was calculated as the ratio of arterial lactate (mmol/L) to serum albumin (g/L). In the context of risk modeling, patients were categorized into survivors and non-survivors based on their discharge status.

### 2.3 Statistical analysis

All statistical analyses were performed using R software (version 4.3.1, The R Foundation for Statistical Computing, Vienna, Austria) and SPSS version 26.0 (IBM Corp, Armonk, NY, USA). Continuous variables were assessed for normality using the Shapiro–Wilk test and visual inspection of histograms. Normally distributed data were expressed as mean ± standard deviation (SD) and compared using the Student's *t*-test. Non-normally distributed variables were presented as median (interquartile range, IQR) and compared using the Mann–Whitney *U* test. Categorical variables were reported as frequencies (percentages) and analyzed using the Chi-square test or Fisher's exact test, as appropriate.

To address the high dimensionality of the data and minimize multicollinearity, the Least Absolute Shrinkage and Selection Operator (LASSO) regression method was employed for variable selection (35, 36). The optimal tuning parameter (lambda, *λ*) was determined using 10-fold cross-validation, selecting the *λ* value that minimized the binomial deviance (*λ*.min) or the value within one standard error of the minimum (*λ*.1se) to ensure model parsimony. Because AIP is derived from triglycerides and HDL-C and LAR is derived from lactate and albumin, pairwise correlations and variance inflation factors (VIFs) were additionally examined for variables retained in the candidate model; a VIF >5 was considered suggestive of clinically relevant collinearity.

Variables identified by LASSO regression were subsequently entered into a multivariable logistic regression model to identify independent predictors of in-hospital mortality. Odds ratios (ORs) with 95% confidence intervals (CIs) were calculated. To facilitate clinical interpretation and ensure model stability, continuous variables exhibiting skewed distributions (e.g., NT-proBNP, hs-cTnI) were log-transformed or standardized (*Z*-score normalization) before modeling.

A prognostic nomogram was constructed based on the final logistic regression model. The discriminative performance of the model was evaluated using the Receiver Operating Characteristic (ROC) curve and the Area Under the Curve (AUC). Calibration was assessed using the Hosmer–Lemeshow goodness-of-fit test, calibration plots (comparing predicted vs. observed probabilities), calibration intercept and slope, and the Brier score; bootstrap resampling (1,000 iterations) was used to obtain internal validation estimates and 95% confidence intervals where appropriate. Furthermore, Decision Curve Analysis (DCA) was performed to quantify the clinical net benefit of the nomogram across a range of threshold probabilities (37). A two-sided *P*-value < 0.05 was considered statistically significant. With 33 outcome events and four predictors retained in the final model, the events-per-variable (EPV) ratio was 8.25, which is below the conventional threshold of 10. Although LASSO-based variable selection was used to limit model complexity and bootstrap resampling was used to assess internal optimism, these procedures do not eliminate overfitting; therefore, the stability of the final model was interpreted cautiously.

## 3 Results

### 3.1 Baseline characteristics of the study population

A total of 207 patients diagnosed with AMI complicated by cardiogenic shock were included in the final analysis. The overall in-hospital mortality rate was 15.9% (33/207). The baseline characteristics of the survivors (*n* = 174) and non-survivors (*n* = 33) are summarized in Table 1. The mean age of the cohort was 66.2 ± 12.8 years, and 67.6% were male. There were no significant differences between the two groups regarding age, sex, smoking status, or comorbidities such as hypertension and diabetes mellitus (*P* > 0.05).

**Table 1:** Baseline characteristics of the study population (*n* = 207).

Variable	Total cohort (*n* = 207)	Survivors (*n* = 174)	Non-survivors (*n* = 33)	Test statistic	*P*-value
Demographics					
Age (years), mean ± SD	66.2 ± 12.8	65.4 ± 12.7	69.3 ± 11.5	*t* = 1.631	0.104
Male sex, *n* (%)	140 (67.6)	118 (67.8)	22 (66.7)	χ² = 0.017	0.897
Comorbidities, *n* (%)					
Hypertension	107 (51.7)	86 (49.4)	21 (63.6)	χ² = 2.243	0.134
Diabetes Mellitus	51 (24.6)	46 (26.4)	5 (15.2)	χ² = 1.903	0.168
Prior Heart Failure	75 (36.2)	57 (32.8)	18 (54.5)	χ² = 5.699	0.017*
AMI presentation and reperfusion					
STEMI, *n* (%)	141 (68.1)	122 (70.1)	19 (57.6)	χ² = 2.008	0.156
NSTEMI, *n* (%)	66 (31.9)	52 (29.9)	14 (42.4)	χ² = 2.008	0.156
Emergency PCI, *n* (%)	116 (56.0)	100 (57.5)	16 (48.5)	χ² = 0.909	0.340
Clinical Parameters					
Heart Rate (bpm), mean ± SD	84.9 ± 27.5	82.9 ± 25.3	95.8 ± 35.1	*t* = 2.522	0.012*
Mean arterial pressure (mmHg), mean ± SD	73.6 ± 15.8	74.2 ± 15.6	70.6 ± 16.9	*t* = −1.130	0.258
Oxygen saturation (%), mean ± SD	96.1 ± 4.9	96.1 ± 5.0	96.2 ± 4.7	*t* = 0.173	0.863
LVEF <40%, *n* (%)	68 (32.9)	48 (27.6)	20 (60.6)	χ² = 13.711	<0.001*
APACHE II score, mean ± SD	12.1 ± 8.8	11.3 ± 7.7	17.1 ± 13.5	*t* = 2.395	0.022*
Laboratory Findings					
Lactate (mmol/L), median (IQR)	2.13 (1.56, 3.92)	2.02 (1.49, 3.50)	5.34 (1.83, 10.39)	*Z* = 3.886	<0.001*
hs-cTnI (ng/mL), mean ± SD	3.89 ± 3.53	3.57 ± 3.36	5.55 ± 3.97	*t* = 2.678	0.011*
NT-proBNP (pg/mL), median (IQR)	2416 (656, 7985)	1963 (486, 6120)	6549 (1299, 18577)	*Z* = 2.893	0.004*
Triglycerides (mmol/L), mean ± SD	1.44 ± 0.95	1.32 ± 0.83	2.09 ± 1.24	*t* = 4.437	<0.001*
HDL-C (mmol/L), mean ± SD	1.01 ± 0.32	1.03 ± 0.33	0.88 ± 0.28	*t* = 2.874	0.006*
Atherogenic Index of Plasma (AIP)	0.10 ± 0.31	0.06 ± 0.30	0.33 ± 0.23	*t* = 5.922	<0.001*
Lactate-to-Albumin Ratio (LAR)	0.10 ± 0.11	0.08 ± 0.08	0.20 ± 0.17	*t* = 4.081	<0.001*
Treatments, *n* (%)					
IABP	25 (12.1)	20 (11.5)	5 (15.2)	Fisher exact	0.562
ECMO	17 (8.2)	10 (5.7)	7 (21.2)	Fisher exact	0.008*
Use of ≥2 vasoactive drugs	103 (49.8)	76 (43.7)	27 (81.8)	χ² = 16.141	<0.001*
CRRT	9 (4.3)	6 (3.4)	3 (9.1)	Fisher exact	0.157
Mechanical Ventilation	79 (38.2)	51 (29.3)	28 (84.8)	χ² = 36.256	<0.001*
Defibrillation	32 (15.5)	18 (10.3)	14 (42.4)	χ² = 21.842	<0.001*

Values are presented as mean ± standard deviation, median (interquartile range), or number (percentage). *P < 0.05 indicates statistical significance. APACHE II score was available in 120 patients (104 survivors and 16 non-survivors). STEMI and NSTEMI were tabulated as mutually exclusive AMI presentation categories. LVEF, left ventricular ejection fraction; hs-cTnI, high-sensitivity cardiac troponin I; NT-proBNP, N-terminal pro-B-type natriuretic peptide; HDL-C, high-density lipoprotein cholesterol; STEMI, ST-segment elevation myocardial infarction; NSTEMI, non-ST-segment elevation myocardial infarction; PCI, percutaneous coronary intervention; IABP, intra-aortic balloon pump; ECMO, extracorporeal membrane oxygenation; CRRT, continuous renal replacement therapy; APACHE II, Acute Physiology and Chronic Health Evaluation II.

Regarding AMI phenotype and early invasive management, 141 patients (68.1%) presented with STEMI and 66 (31.9%) with NSTEMI, while 116 patients (56.0%) underwent emergency PCI. Compared with survivors, non-survivors more frequently required ECMO (21.2% vs. 5.7%, *P* = 0.008), use of two or more vasoactive drugs (81.8% vs. 43.7%, *P* < 0.001), mechanical ventilation (84.8% vs. 29.3%, *P* < 0.001), and defibrillation (42.4% vs. 10.3%, *P* < 0.001). STEMI/NSTEMI presentation and emergency PCI did not differ significantly between the two groups (all *P* > 0.05). Among patients with available APACHE II data (*n* = 120), non-survivors also had higher APACHE II scores (17.1 ± 13.5 vs. 11.3 ± 7.7, *P* = 0.022).

Compared to survivors, non-survivors presented with significantly more severe hemodynamic instability and metabolic derangements. Specifically, non-survivors had higher heart rates (95.8 ± 35.1 vs. 82.9 ± 25.3 bpm, *P* = 0.012) and significantly elevated levels of lactate (median 5.34 vs. 2.02 mmol/L, *P* < 0.001). Markers of myocardial injury and cardiac dysfunction were markedly worse in the non-survivor group, as evidenced by higher hs-cTnI (5.55 ± 3.97 vs. 3.57 ± 3.36 ng/mL, *P* = 0.011), higher NT-proBNP (median 6549 vs. 1963 pg/mL, *P* = 0.004), and a higher prevalence of LVEF <40% (60.6% vs. 27.6%, *P* < 0.001).

Regarding the biomarkers of interest, non-survivors exhibited significantly higher triglyceride levels (2.09 ± 1.24 vs. 1.32 ± 0.83 mmol/L, *P* < 0.001) and lower HDL-C levels (0.88 ± 0.28 vs. 1.03 ± 0.33 mmol/L, *P* = 0.006). Consequently, the Atherogenic Index of Plasma (AIP) was significantly elevated in the non-survivor group (0.33 ± 0.23 vs. 0.06 ± 0.30, *P* < 0.001). Similarly, the Lactate-to-Albumin Ratio (LAR) was significantly higher in non-survivors (0.20 ± 0.17 vs. 0.08 ± 0.08, *P* < 0.001), driven by both hyperlactatemia and hypoalbuminemia. Additionally, non-survivors had higher inflammatory markers (hs-CRP) and required more intensive treatments, including mechanical ventilation (84.8% vs. 29.3%, *P* < 0.001) and electrical defibrillation (42.4% vs. 10.3%, *P* < 0.001).

### 3.2 Variable selection using LASSO regression

To identify the most robust predictors of mortality and avoid overfitting due to the relatively small number of events (33 deaths), we utilized LASSO regression analysis on 18 potentially significant variables identified from the univariable analysis. The LASSO coefficient profiles were generated against the log(lambda) sequence (Figure 1). Using 10-fold cross-validation, the optimal lambda (*λ*) was selected based on the minimum binomial deviance criteria (Figure 2). This process reduced the dimensionality of the data, identifying six non-zero coefficient variables as candidate predictors: Mechanical Ventilation (MV), LVEF <40%, AIP, LAR, Triglycerides (TG), and hs-CRP. The standardized importance of these selected variables is shown in Figure 3. Notably, unlike standard stepwise selection, LASSO penalizes coefficients, effectively shrinking less important variables to zero; nevertheless, because only 33 outcome events occurred, the selected predictors and final model should be interpreted cautiously.

**Figure 1:**
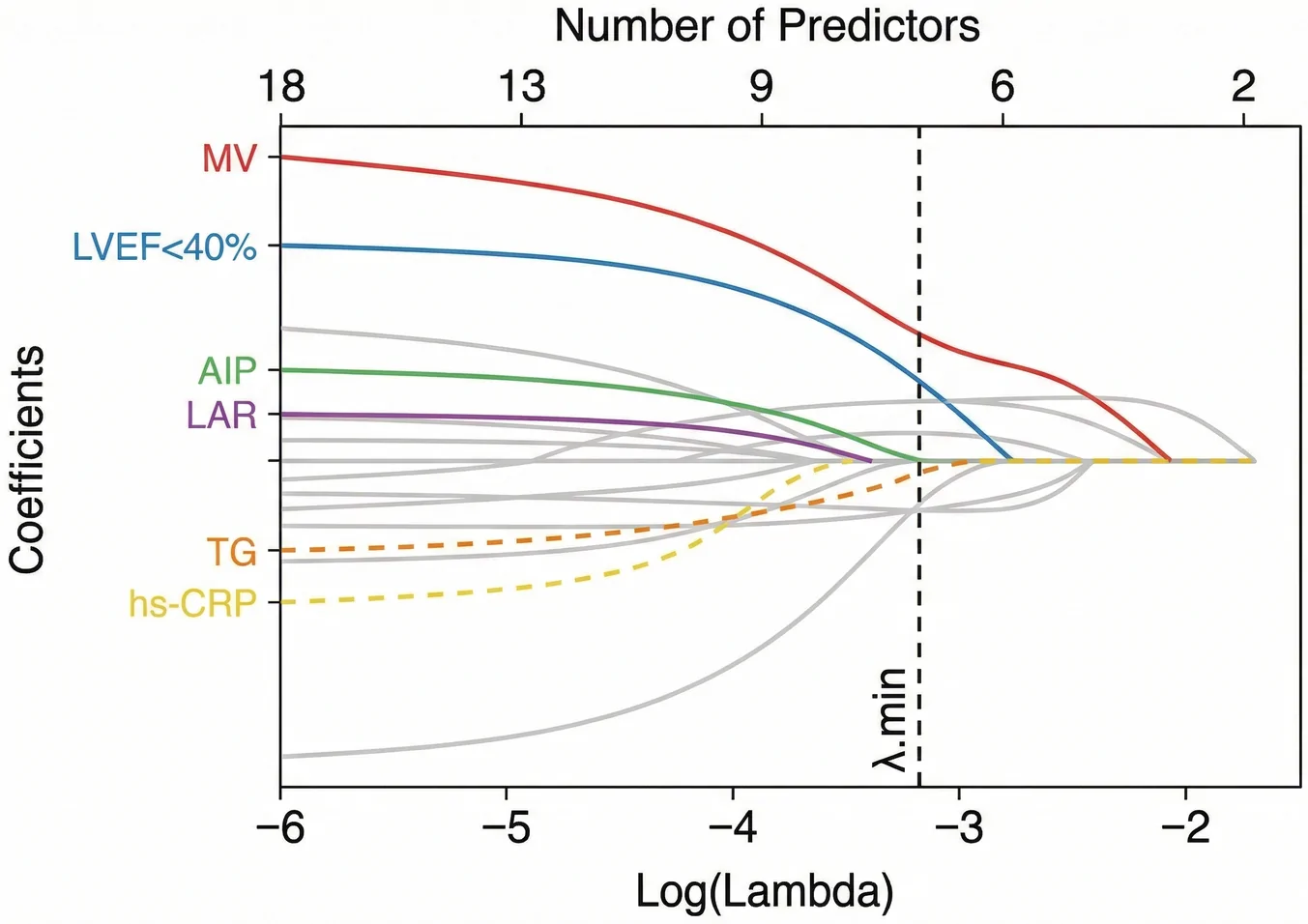
LASSO coefficient profiles of the 18 potential predictors. Each curve represents one candidate variable, and the *x*-axis shows log(lambda), where larger lambda values impose stronger shrinkage. As lambda increases, coefficients of less informative variables shrink toward zero. Variables with non-zero coefficients at the selected lambda were retained as candidate predictors for subsequent multivariable modeling.

**Figure 2:**
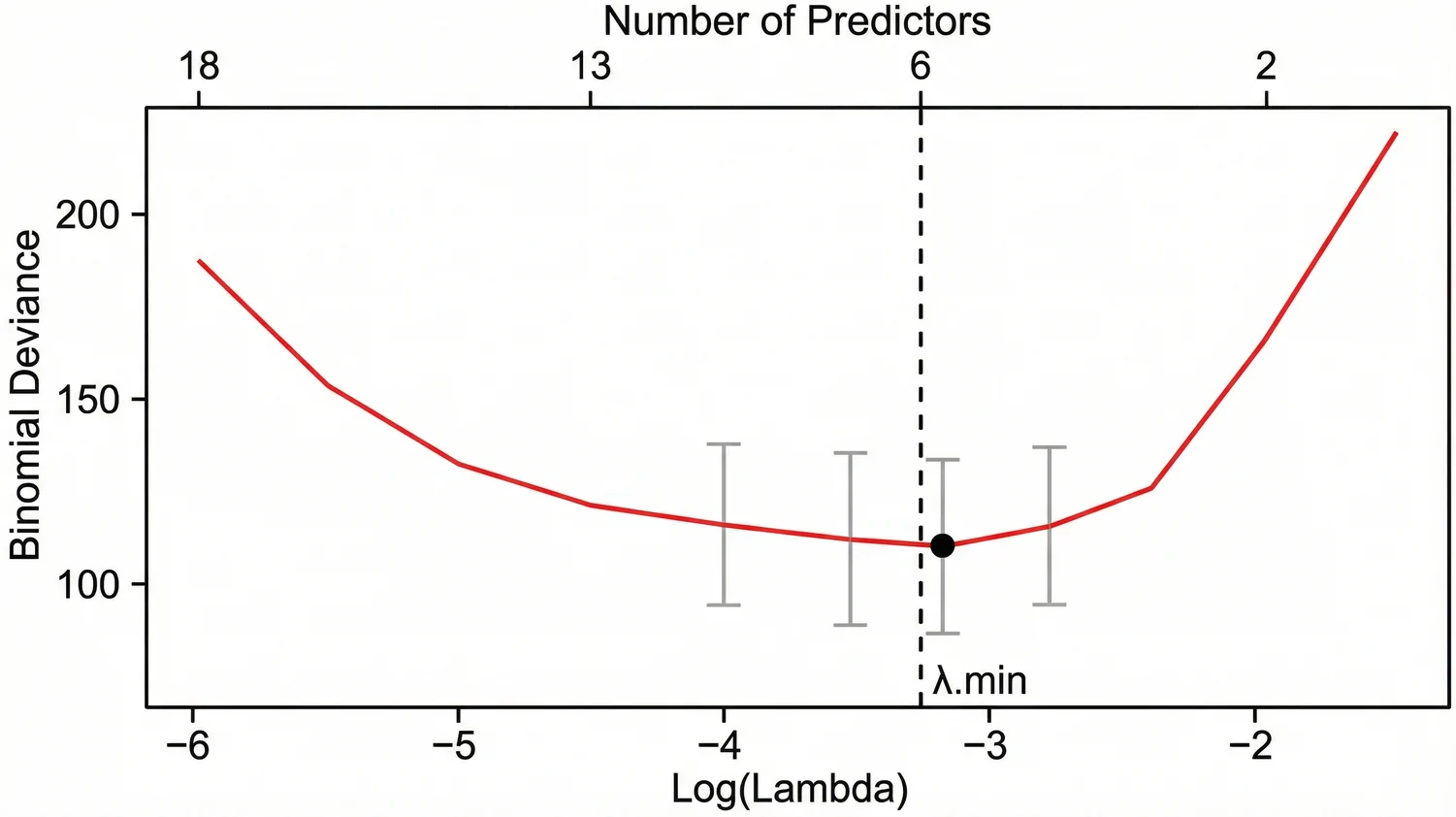
Selection of the optimal tuning parameter (lambda) in the LASSO model. The plot displays binomial deviance from 10-fold cross-validation versus log(lambda). A dotted vertical line indicates lambda.min, which gives the minimum cross-validated deviance and corresponds to six retained predictors. The selected lambda was used for candidate-variable selection.

**Figure 3:**
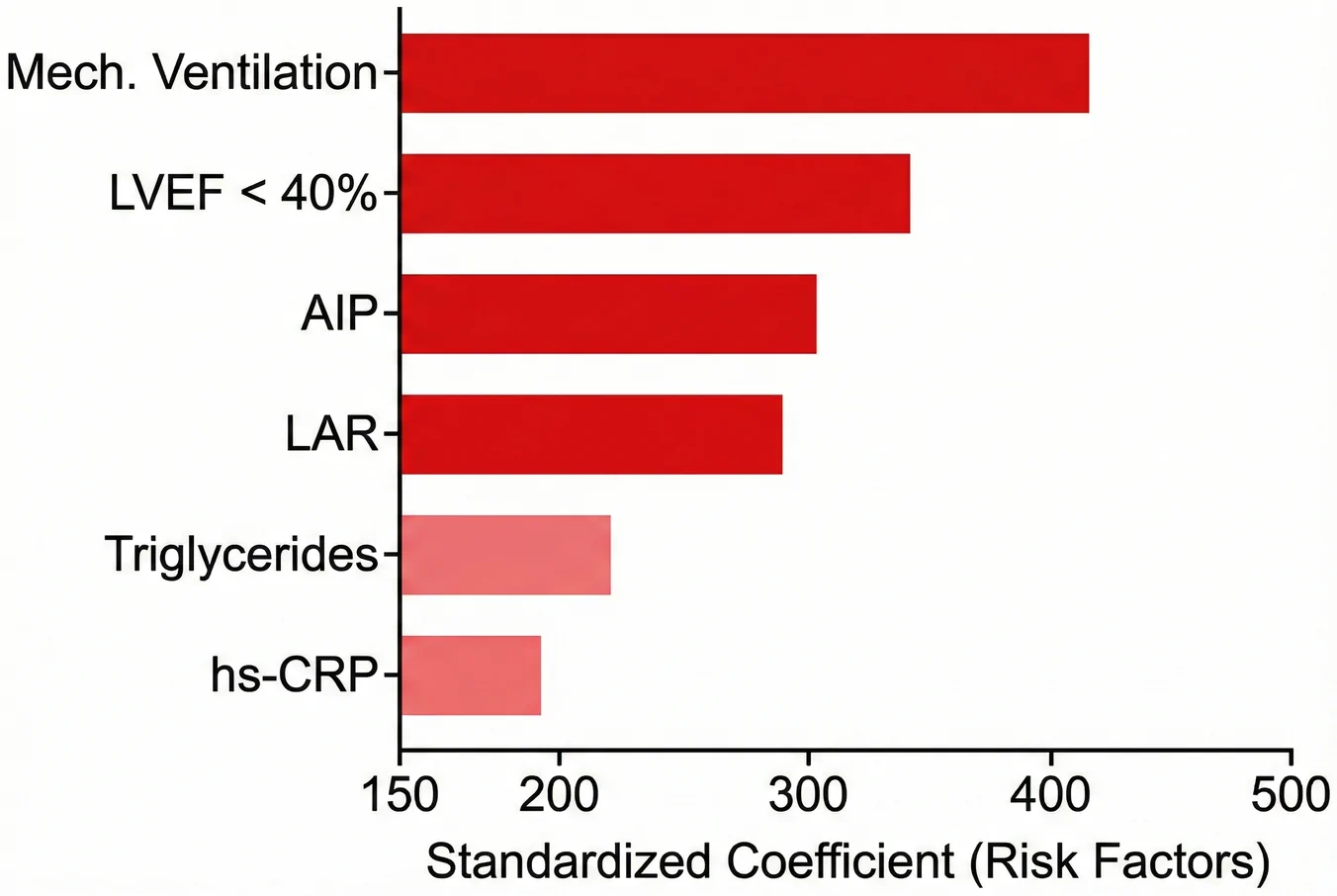
Importance of selected variables. Bar chart showing the standardized coefficients of the variables retained by LASSO regression. Longer bars indicate greater relative contribution to the penalized model, while positive values indicate variables associated with higher mortality risk.

### 3.3 Multivariable logistic regression analysis

The six variables identified by LASSO were first evaluated as candidate predictors. Triglycerides and hs-CRP were not retained in the final parsimonious nomogram because they did not improve model parsimony and overlapped with the composite indices. The final multivariable logistic regression model, presented in Table 2, retained four independent predictors of in-hospital mortality. Mechanical ventilation was the strongest binary predictor, associated with an approximately 10-fold increase in mortality risk (OR = 10.10, 95% CI: 3.09–33.09, *P* < 0.001). Patients with severe left ventricular dysfunction (LVEF <40%) had a higher risk of death (OR = 3.49, 95% CI: 1.23–9.90, *P* = 0.019).

**Table 2:** Multivariable logistic regression analysis identifying independent predictors of in-hospital mortality.

Variable	*β* coefficient	Standard error	Wald χ²	Odds ratio (95% CI)	*P*-value
Mechanical Ventilation (Yes vs. No)	2.313	0.605	14.60	10.10 (3.09–33.09)	<0.001*
LVEF <40% (Yes vs. No)	1.248	0.533	5.49	3.49 (1.23–9.90)	0.019*
AIP (per 1-SD increase)	1.458	0.337	18.67	4.30 (2.22–8.32)	<0.001*
LAR (per 1-SD increase)	0.632	0.227	7.77	1.88 (1.21–2.93)	0.005*
Constant	−4.258	0.662	41.37	0.014 (0.004–0.050)	<0.001*

Variables were initially screened using LASSO regression. The final parsimonious model retained the four independent predictors used to construct the nomogram. Continuous variables (AIP and LAR) were standardized (Z-score normalization) before model entry; “per 1-SD increase” indicates the change in odds for every standard deviation increase in the biomarker. CI, confidence interval.

The symbol * indicates statistical significance: *P < 0.05.

Crucially, the novel biomarkers maintained their independent predictive value. Elevated AIP was significantly associated with increased mortality (OR = 4.30 per 1-SD increase, 95% CI: 2.22–8.32, *P* < 0.001). Similarly, a higher LAR independently predicted poor outcomes (OR = 1.88 per 1-SD increase, 95% CI: 1.21–2.93, *P* = 0.005). Collinearity assessment showed that AIP was strongly correlated with triglycerides by design (Pearson *r* = 0.834; Spearman *ρ* = 0.879), whereas LAR was strongly correlated with lactate (Spearman *ρ* = 0.967). In the six-variable candidate set, however, VIF values were acceptable for mechanical ventilation (1.21), LVEF <40% (1.12), AIP (3.37), LAR (1.21), triglycerides (3.34), and hs-CRP (1.07), suggesting that severe multicollinearity was not present.

### 3.4 Model performance and discrimination

The discriminatory power of the individual predictors and the combined model was evaluated using ROC curve analysis (Figure 4 and Table 3). As detailed in Table 3, the Area Under the Curve (AUC) values for the individual predictors were: Mechanical Ventilation (0.778), AIP (0.777), LAR (0.735), and LVEF <40% (0.665). The combined prediction model, incorporating MV, LVEF <40%, AIP, and LAR, achieved an AUC of 0.909 (bootstrap 95% CI: 0.853–0.957), indicating good-to-excellent discrimination in the derivation cohort. Bootstrap paired comparisons confirmed that the combined model was statistically superior to any single predictor alone (Table 3, all *P* < 0.001). The model exhibited a sensitivity of 78.8% and a specificity of 86.2% at the optimal cutoff point.

**Figure 4:**
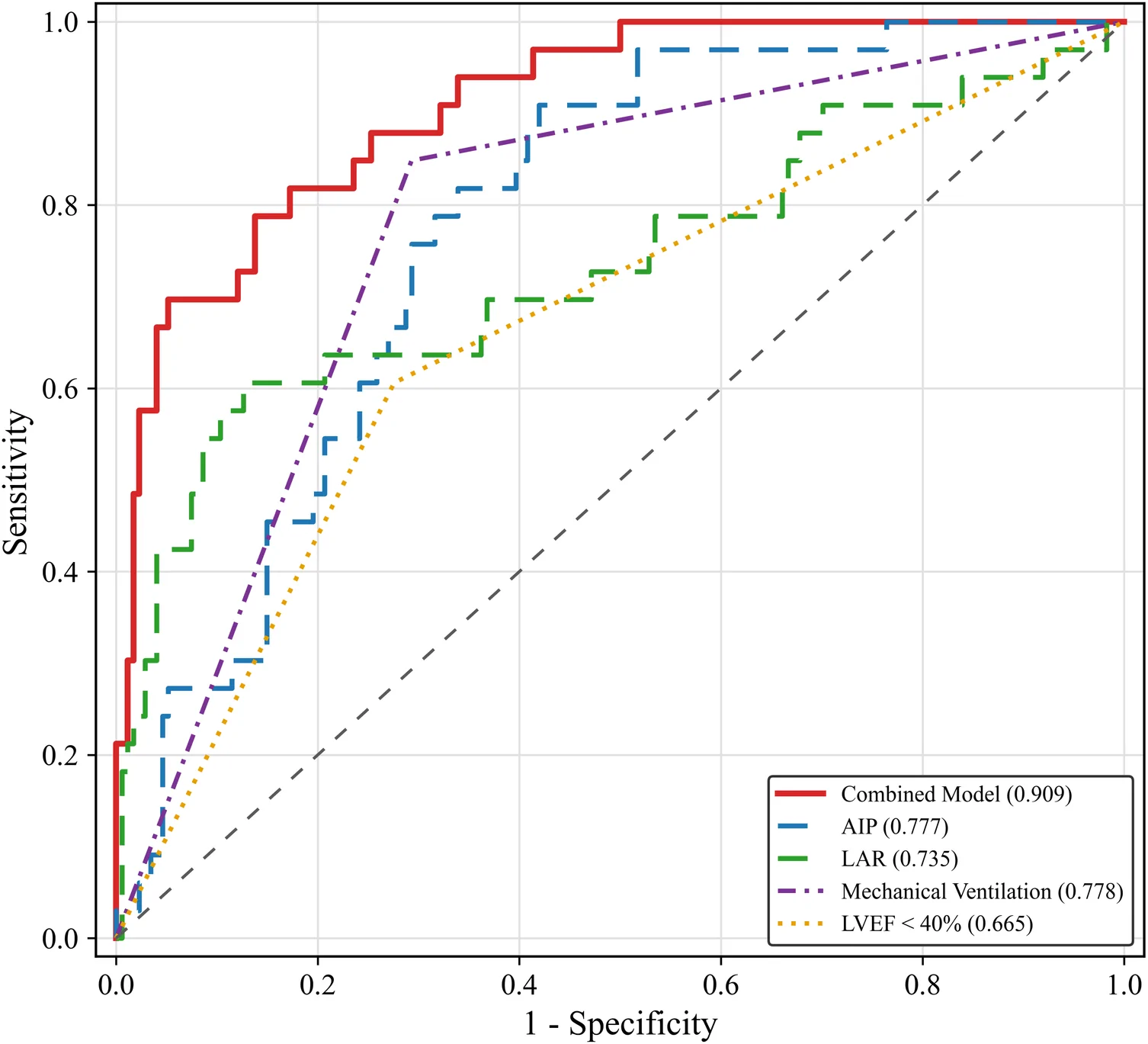
Receiver operating characteristic (ROC) curves. ROC analysis comparing the discriminatory ability of the key predictors included in the final nomogram (Mechanical Ventilation, AIP, LAR, and LVEF <40%) and the Combined Prediction Model for in-hospital mortality. The Combined Model (AUC = 0.909) showed superior internal discrimination compared with any single predictor, but this finding requires external validation.

**Table 3:** Comparative analysis of model performance (AUC) for in-hospital mortality prediction.

Predictor/model	AUC	Standard error	95% confidence interval	*P*-value vs. combined model
Individual Predictors				
Mechanical Ventilation	0.778	0.042	0.695–0.860	<0.001
Atherogenic Index of Plasma (AIP)	0.777	0.037	0.704–0.849	<0.001
Lactate-to-Albumin Ratio (LAR)	0.735	0.056	0.625–0.844	<0.001
LVEF <40%	0.665	0.053	0.560–0.770	<0.001
Integrated Model				
Combined Prediction Model^a^	0.909	0.026	0.853–0.957	-

a The Combined Prediction Model incorporates Mechanical Ventilation, LVEF <40%, AIP, and LAR. AUC comparisons were assessed using bootstrap paired differences. The combined model demonstrated superior discrimination compared to any single predictor alone. AUC, area under the curve; LVEF, left ventricular ejection fraction.

### 3.5 Nomogram construction and validation

To facilitate clinical application, a prognostic nomogram was developed based on the independent predictors: MV, LVEF <40%, AIP, and LAR (Figure 5). In this nomogram, each predictor is assigned a score on a point scale. By summing the scores for all variables and locating the total score on the total point scale, clinicians can estimate the individual probability of in-hospital mortality for a patient with AMICS.

**Figure 5:**
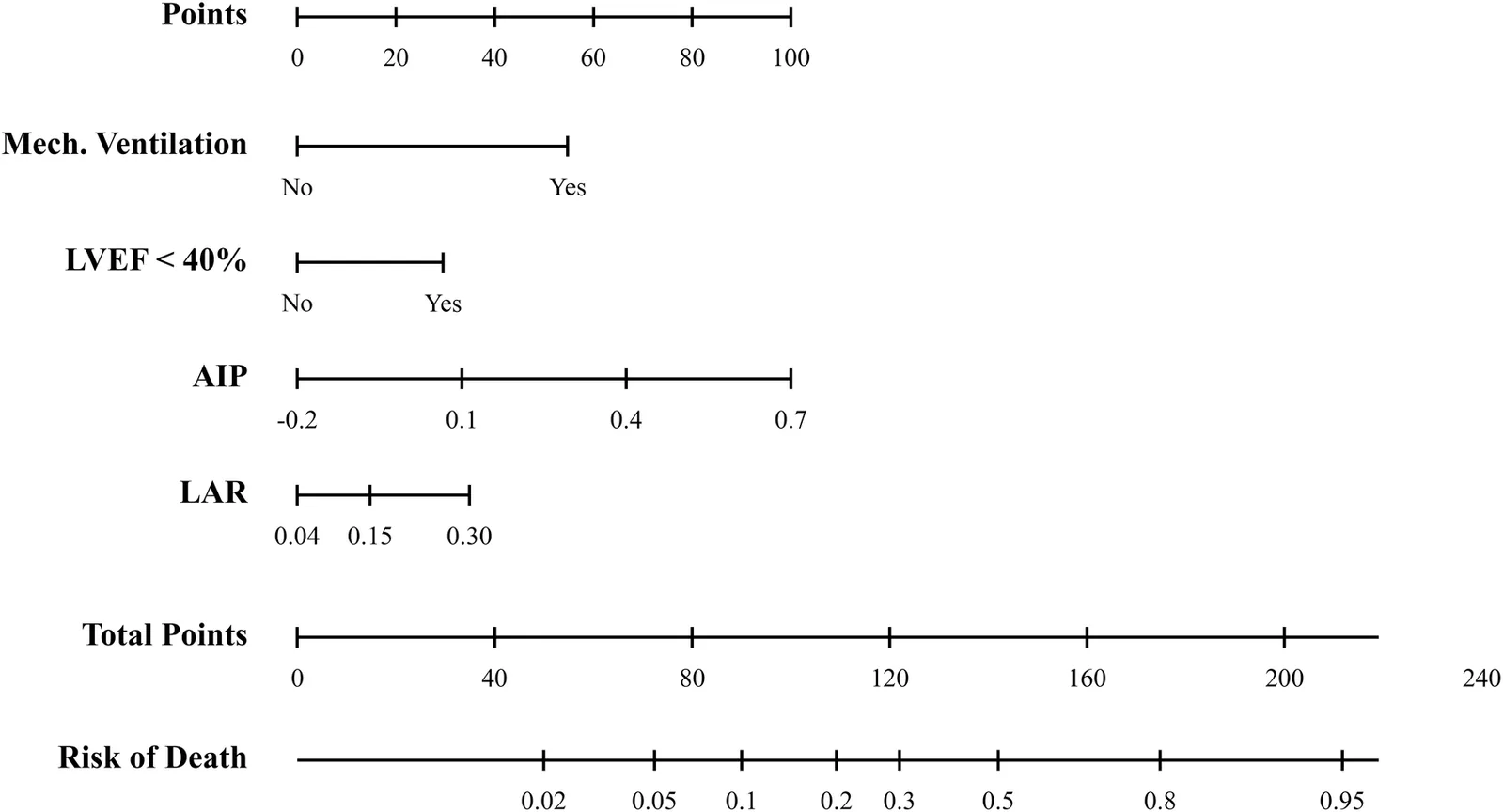
Nomogram for predicting in-hospital mortality in AMICS patients. To use the nomogram, locate the patient's value for each variable on the corresponding axis. Draw a vertical line upward to the “Points” axis to determine the score for that variable. Sum the scores for all variables to get the “Total Points”. Locate the total points on the bottom scale to determine the predicted probability of in-hospital mortality.

The calibration of the model was assessed using the Hosmer–Lemeshow test, which yielded a non-significant *P*-value (χ^2^ = 4.04, *P* = 0.853), suggesting acceptable agreement between predicted and observed probabilities. The calibration plot based on deciles of predicted risk (Figure 6) demonstrated close apparent agreement with the ideal reference line, with a mean absolute error of 0.024. Additional calibration-related metrics are shown in Table 4: the Brier score was 0.077 (bootstrap 95% CI: 0.052–0.103), the calibration intercept was 0.00 (95% CI: −0.52 to 0.67), and the calibration slope was 1.00 (95% CI: 0.75–1.53). The Hosmer–Lemeshow test and Brier score suggested acceptable apparent calibration; however, the bootstrap confidence intervals for the calibration intercept and slope remained relatively wide, indicating uncertainty in calibration stability. Therefore, these findings should be interpreted as internal validation only and do not replace external validation. To facilitate descriptive clinical interpretation, patients were stratified *post hoc* into four categories according to nomogram-predicted risk: <10% (*n* = 137), 10%–30% (*n* = 31), 30%–60% (*n* = 19), and ≥60% (*n* = 20). The corresponding observed numbers of in-hospital deaths in these categories were 4 (2.9%), 4 (12.9%), 9 (47.4%), and 16 (80.0%), respectively, illustrating a stepwise risk ranking within this derivation cohort. These categories were used for descriptive interpretation only and should not be regarded as externally validated clinical decision thresholds.

**Figure 6:**
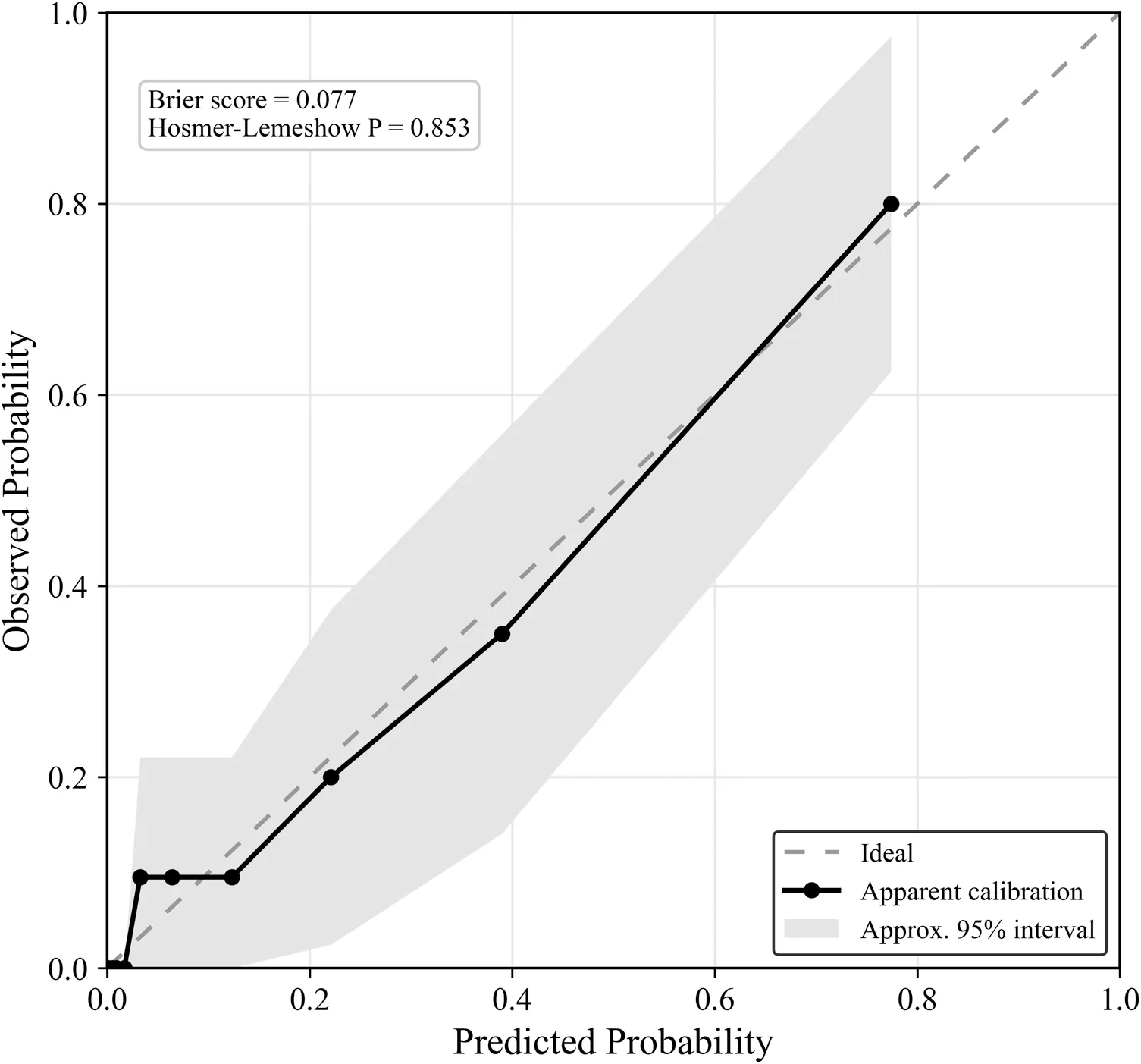
Calibration plot of the nomogram. The *x*-axis represents the predicted probability of in-hospital mortality, and the *y*-axis represents the observed probability. The diagonal dotted line represents ideal calibration. The solid line represents apparent calibration across deciles of predicted risk, and the shaded area indicates an approximate 95% interval. Calibration results should be interpreted cautiously because confidence intervals for calibration metrics were relatively wide and no external validation cohort was available.

**Table 4:** Calibration-related metrics for the combined prediction model.

Calibration metric	Estimate	95% confidence interval/*P*-value	Method
Hosmer–Lemeshow χ²	4.04	*P* = 0.853	Goodness-of-fit test
Mean absolute error	0.024	Not applicable	Grouped calibration plot
Brier score	0.077	0.052–0.103	1,000 bootstrap resamples
Calibration intercept	0.00	−0.52–0.67	1,000 bootstrap resamples
Calibration slope	1.00	0.75–1.53	1,000 bootstrap resamples

The Hosmer–Lemeshow test does not conventionally provide a confidence interval; bootstrap confidence intervals are therefore reported for Brier score, calibration intercept, and calibration slope. Ideal values are 0 for the calibration intercept and 1 for the calibration slope; deviations from these values and wide confidence intervals should be interpreted cautiously. All calibration metrics were derived from 207 patients, among whom 33 (15.9%) experienced the outcome event (in-hospital death).

### 3.6 Clinical utility

Decision Curve Analysis (DCA) was performed to evaluate the exploratory clinical utility of the nomogram (Figure 7). DCA suggested that the nomogram provided an apparent net benefit compared with the treat-all and treat-none strategies across the threshold range shown in the DCA plot. Given the single-center design and limited number of outcome events, this finding should be interpreted as exploratory and should be confirmed in external validation cohorts before clinical implementation.

**Figure 7:**
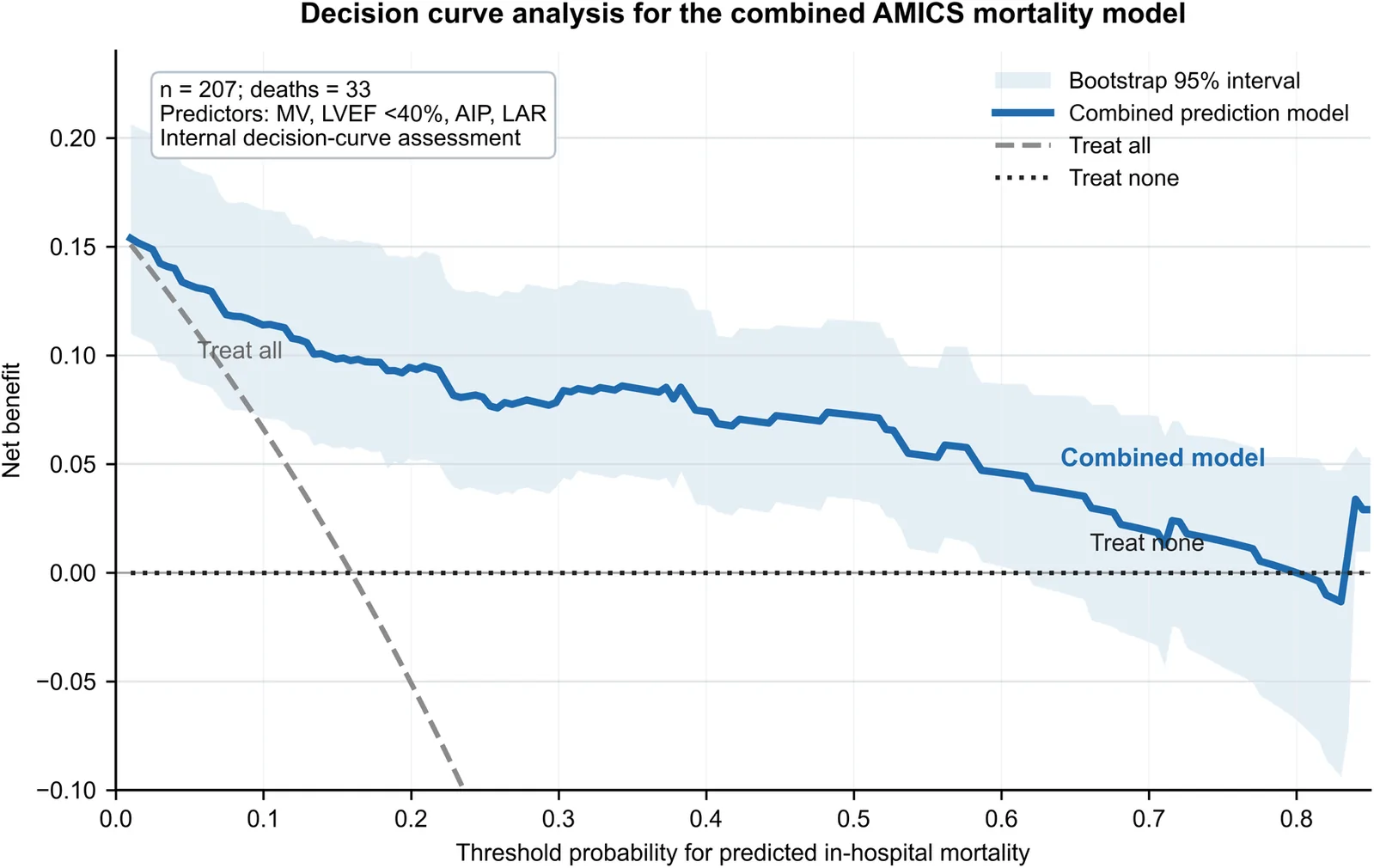
Decision curve analysis for the combined prediction model. The *x*-axis indicates the threshold probability for predicting in-hospital mortality, and the *y*-axis indicates net benefit. The combined model is compared with the treat-all and treat-none strategies. The analysis suggests apparent clinical net benefit across the displayed threshold range, but external validation is required before clinical implementation.

## 4 Discussion

In this retrospective cohort study of 207 patients with acute myocardial infarction complicated by cardiogenic shock (AMICS), we demonstrated that the Atherogenic Index of Plasma (AIP) and the Lactate-to-Albumin Ratio (LAR) were independently associated with in-hospital mortality. Furthermore, we developed an internally validated nomogram incorporating these two biomarkers alongside Mechanical Ventilation (MV) and Left Ventricular Ejection Fraction (LVEF) <40%. The model exhibited good-to-excellent discrimination (AUC = 0.909) and acceptable calibration in the derivation cohort, outperforming individual biomarkers in risk stratification. To our knowledge, this is the first study to integrate a lipid metabolic index with an inflammatory-perfusion marker to predict outcomes in an AMICS population, although the findings should be considered exploratory until externally validated.

### 4.1 The prognostic value of AIP in AMICS: beyond atherogenesis

The Atherogenic Index of Plasma (AIP), calculated as log(TG/HDL-C), has traditionally been utilized as a surrogate marker for plasma atherogenicity and the particle size of small dense low-density lipoprotein (sdLDL) (38). While its role in predicting long-term cardiovascular events in coronary artery disease (CAD) is well-established (39, 40), its significance in the acute phase of AMICS has been largely unexplored. Our study found that non-survivors had significantly higher AIP levels compared to survivors, and AIP remained an independent predictor of mortality after adjusting for confounders (OR = 4.30 per SD increase).

The mechanism underlying the association between elevated AIP and poor prognosis in AMICS is likely multifaceted. First, AIP is a robust marker of insulin resistance and metabolic syndrome (13). In the setting of acute physiological stress like cardiogenic shock, insulin resistance exacerbates hyperglycemia and impairs myocardial glucose uptake, forcing the ischemic heart to rely on less efficient fatty acid oxidation, thereby worsening myocardial energy deficits (41). Second, elevated triglycerides and low HDL-C levels, which drive a high AIP, are associated with endothelial dysfunction and impaired nitric oxide bioavailability (42). In CS, where microcirculatory dysfunction is a key driver of multi-organ failure (43), this pre-existing or acute endothelial impairment may precipitate irreversible shock. Third, HDL-C possesses anti-inflammatory and antioxidant properties (44). The significantly lower HDL-C levels observed in our non-survivor group (0.88 vs. 1.03 mmol/L) suggest a depleted antioxidant reserve, leaving the myocardium vulnerable to oxidative stress and reperfusion injury. Thus, AIP in AMICS likely reflects a “metabolic fragility” phenotype that tolerates hemodynamic collapse poorly.

### 4.2 LAR as a composite marker of hypoperfusion and inflammation

Lactate is the canonical marker of tissue hypoxia and anaerobic metabolism in shock, though its levels can be influenced by factors such as hepatic clearance and adrenergic drive (45). Albumin, conversely, is a well-established negative acute-phase reactant; its serum levels drop rapidly during critical illness due to increased capillary permeability and cytokine-mediated suppression of hepatic synthesis (46). The Lactate-to-Albumin Ratio (LAR) integrates these two core pathophysiological processes: tissue hypoperfusion (reflected in the numerator) and the severity of systemic inflammation/nutritional depletion (reflected in the denominator).

Our findings align with recent studies in sepsis and heart failure, which identified LAR as a useful prognostic marker compared to lactate alone (24, 47). Similarly, in critically ill patients with heart failure, a higher LAR has been associated with worse clinical outcomes (29). In our AMICS cohort, LAR was markedly elevated in non-survivors (0.20 vs. 0.08) and emerged as a strong independent predictor of mortality (OR = 1.88 per SD increase). The potential advantage of LAR over isolated lactate measurement stems from its ability to concurrently capture tissue hypoperfusion and hypoalbuminemia, the latter reflecting inflammation, nutritional reserve, capillary leakage, and hepatic synthetic stress. A high LAR, therefore, may indicate a patient who is not only hypoperfused but also physiologically vulnerable to prolonged shock. This interpretation remains mechanistic and hypothesis-generating rather than proof of causality.

### 4.3 Clinical implications of the combined model

Current risk stratification tools for acute myocardial infarction complicated by cardiogenic shock (AMICS), such as the IABP-SHOCK II score, rely heavily on hemodynamic variables and classic clinical risk factors like age and prior stroke (48). While these tools demonstrate effective predictive performance (49, 50), they often overlook the critical metabolic and inflammatory axes of the shock pathophysiology (51). Our study proposes a novel “Metabolic-Inflammatory” risk stratification model. The integration of the Atherogenic Index of Plasma (AIP) and the Lactate-to-Albumin Ratio (LAR) into a nomogram alongside mechanical ventilation (MV) and left ventricular ejection fraction (LVEF) <40% yielded a high area under the curve (AUC) of 0.909, suggesting good-to-excellent apparent discriminatory performance in this single-center derivation cohort. Recent ACS data showing the prognostic relevance of composite inflammatory-nutritional indices such as PIV and HALP further support the broader concept that multidimensional biomarkers may complement conventional risk assessment (52). A conceptual comparison of the present nomogram with the established IABP-SHOCK II and CardShock risk scores is provided in Supplementary Table S1 (53, 54). Whereas these established scores rely primarily on demographic, hemodynamic, and procedural variables and have been externally validated, the present model additionally captures the lipid-metabolic and hypoperfusion-inflammatory dimensions of AMICS pathophysiology, but it remains internally validated only.

The clinical utility of this model is potentially meaningful but should be interpreted with appropriate caution. Mechanical ventilation and severe left ventricular dysfunction are established markers of shock severity, reflecting respiratory failure and pump failure, respectively. By adding AIP and LAR, the model incorporates the patient's metabolic reserve and systemic hypoperfusion-inflammatory burden. AIP is a well-established marker of atherogenic dyslipidemia and inflammation, correlating with cardiovascular risk and plaque instability (13, 55). LAR serves as an integrative marker of lactate burden and albumin depletion rather than a direct leukocyte-based inflammatory index. For instance, a patient with borderline hemodynamics but very high AIP and LAR may warrant closer monitoring and earlier reassessment for escalation of care; however, the present data do not prove that biomarker-guided escalation improves outcomes.

The inclusion of mechanical ventilation in the final model also requires careful interpretation. Mechanical ventilation is not a causal biological determinant of mortality in the same way as a metabolic or inflammatory biomarker; rather, it reflects advanced respiratory failure, pulmonary edema, altered consciousness, or the need for intensive supportive care. We retained this variable because it was selected by LASSO and independently associated with mortality, and because it improved short-term risk discrimination in the hospitalized cohort. Nevertheless, its presence may limit the use of the nomogram as a purely baseline pre-intervention tool. The model is therefore best interpreted as an early in-hospital risk stratification aid after initial clinical assessment, not as a standalone triage algorithm before therapeutic decisions have been made.

### 4.4 Limitations

Several limitations of this study must be acknowledged. First, this was a single-center retrospective study with a relatively small sample size (*n* = 207) and only 33 in-hospital deaths. Although LASSO-based selection and a parsimonious final model were used to limit model complexity, and bootstrap resampling was used to assess internal optimism, the events-per-variable ratio of 8.25 indicates that model instability and overfitting remain possible. Therefore, the relatively high AUC should be interpreted cautiously. Second, no independent external validation cohort was available, which limits the generalizability and clinical readiness of the nomogram. Third, the observed in-hospital mortality rate of 15.9% was lower than the 40%–50% mortality commonly reported in contemporary AMICS registries. This discrepancy may relate to the study's single-center CCU setting, exclusion of out-of-hospital cardiac arrest with severe hypoxic brain injury, exclusion of non-AMI shock and severe non-cardiac confounders, and possible under-representation of the most extreme SCAI stage E phenotype. Fourth, SCAI shock stage was not prospectively recorded, and therefore the severity distribution of shock could not be directly compared with contemporary SCAI-based cohorts (17, 18). Fifth, although we added available information on STEMI/NSTEMI presentation, emergency PCI, IABP, ECMO, vasoactive drug use, CRRT, and APACHE II score, detailed infarct localization, culprit-vessel anatomy, time from symptom onset to reperfusion, and procedural PCI details were not consistently available in the retrospective dataset. Sixth, AIP and LAR were measured only at admission; dynamic changes during hospitalization might provide additional prognostic information. Finally, residual confounding from unmeasured variables and variation in the timing of emergency therapies, including vasopressor initiation before blood sampling in some unstable patients, cannot be excluded. Future multicenter prospective studies with standardized SCAI staging, angiographic variables, serial biomarker measurements, and external validation are needed to confirm these findings.

## 5 Conclusion

In conclusion, elevated AIP and LAR were independently associated with in-hospital mortality in patients with AMICS. A nomogram integrating AIP, LAR, mechanical ventilation, and LVEF <40% showed good-to-excellent apparent/internal discrimination and acceptable calibration in this single-center cohort. Because mechanical ventilation may become available only after some early therapeutic decisions have already been made, its inclusion means that the nomogram is best applied as an early in-hospital reassessment tool after initial clinical evaluation rather than as a pre-treatment triage instrument. These findings suggest that lipid-metabolic and hypoperfusion-inflammatory biomarkers may complement conventional severity markers for early risk stratification. However, given the limited number of events, lack of external validation, absence of SCAI-stage stratification, and unexpectedly low observed mortality, the nomogram should be considered preliminary and requires prospective multicenter validation before routine clinical use.

## Data Availability

The original contributions presented in the study are included in the article/Supplementary Material, further inquiries can be directed to the corresponding author.
