# Supplementary material for: Prognostic value of the atherogenic index of plasma combined with lactate-to-albumin ratio for in-hospital mortality in patients with acute myocardial infarction complicated by cardiogenic shock
Source: Front Cardiovasc Med. 2026 Sep 14;13:1816471. doi: 10.3389/fcvm.2026.1816471 (PMC13616785; doi:10.3389/fcvm.2026.1816471)
Supplement: Supplementary file 1 [file Table1.docx]

**Supplementary Table S1. Conceptual Comparison of the Present Nomogram with Established Risk Scores for Cardiogenic Shock Complicating Acute Myocardial Infarction**

| **Feature** | **IABP-SHOCK II score** | **CardShock score** | **Present nomogram** |
| --- | --- | --- | --- |
| Derivation cohort | Patients with complete data from the IABP-SHOCK II trial | CardShock study (multinational European registry) | Single-center CCU cohort (Renmin Hospital, Hubei University of Medicine) |
| Study design | RCT-derived (Germany) | Prospective observational (9 European countries) | Retrospective observational |
| Population | AMI-related cardiogenic shock | Cardiogenic shock of ACS and non-ACS aetiologies | AMI complicated by cardiogenic shock (AMICS) |
| Sample size | 480 in the development cohort; external validation cohorts: n=98 and n=137 | 219 | 207 (33 events) |
| Outcome | 30-day all-cause mortality | Short-term (in-hospital) mortality | In-hospital all-cause mortality |
| Predictor variables | Age >73 years, prior stroke, glucose >10.6 mmol/L, creatinine >132.6 umol/L, TIMI flow grade <3 after PCI, lactate >5 mmol/L | Age, previous MI, prior CABG, confusion, ACS aetiology, LVEF, systolic blood pressure, lactate, eGFR | Mechanical ventilation, LVEF <40%, AIP, LAR |
| Includes lipid-metabolic biomarkers | No | No | Yes (AIP: lipid metabolism / insulin resistance axis) |
| Includes hypoperfusion-inflammatory biomarkers | Partially (lactate only) | Partially (lactate only) | Yes (LAR: lactate-to-albumin ratio) |
| Includes respiratory support status | No | No | Yes |
| Reported discrimination (AUC) | 0.79 (registry validation); 0.73 (CardShock external validation) | 0.85 in the derivation cohort; approximately 0.74 in an external ACS-related cardiogenic shock cohort | 0.909 (internal, bootstrap 95% CI 0.853-0.957) |
| External validation | Validated in external cohorts | Externally validated; performance varies across cohorts and shock aetiologies | Not yet externally validated (hypothesis-generating) |
| Primary intended use | Short-term mortality risk stratification in infarct-related cardiogenic shock, incorporating post-PCI TIMI flow | Early risk stratification in broad CS populations | Exploratory early in-hospital risk stratification after initial assessment |

Note: The comparison is conceptual and is based on the original derivation publications and subsequent validation studies [8, 9, 49, 53, 54]. The reported AUC values were obtained from different study populations and outcomes and should not be interpreted as head-to-head evidence of comparative superiority. AUC: area under the receiver operating characteristic curve; AMI: acute myocardial infarction; ACS: acute coronary syndrome; CS: cardiogenic shock; AMICS: acute myocardial infarction complicated by cardiogenic shock; AIP: Atherogenic Index of Plasma; LAR: Lactate-to-Albumin Ratio; LVEF: left ventricular ejection fraction; eGFR: estimated glomerular filtration rate; TIMI: Thrombolysis in Myocardial Infarction; PCI: percutaneous coronary intervention; MI: myocardial infarction; CABG: coronary artery bypass grafting; CCU: coronary care unit; RCT: randomized controlled trial.
